# Physiological and molecular responses to high-temperature stress at anthesis in Brazilian flooded rice

**DOI:** 10.1093/aobpla/plaf043

**Published:** 2025-08-23

**Authors:** Silmara da Luz Correia, Kanjana Laosuntisuk, Jigar Desai, Paulo Regis Ferreira da Silva, Carla Andrea Delatorre, Colleen J Doherty

**Affiliations:** Department of Crop Sciences, Federal University of Rio Grande do Sul (UFRGS), Porto Alegre, RS 90010-150, Brazil; Bayer, Chapadão do Sul, MS, Brazil; Department of Molecular and Structural Biochemistry, North Carolina State University, 120 Broughton Dr. CB 7622, Raleigh, NC 27695-7622, United States; Faculty of Medicine Siriraj Hospital, Mahidol University, Chang Wat Nakhon Pathom 73170, Thailand; Department of Molecular and Structural Biochemistry, North Carolina State University, 120 Broughton Dr. CB 7622, Raleigh, NC 27695-7622, United States; Department of Crop Sciences, Federal University of Rio Grande do Sul (UFRGS), Porto Alegre, RS 90010-150, Brazil; Department of Crop Sciences, Federal University of Rio Grande do Sul (UFRGS), Porto Alegre, RS 90010-150, Brazil; Department of Molecular and Structural Biochemistry, North Carolina State University, 120 Broughton Dr. CB 7622, Raleigh, NC 27695-7622, United States; Form & Function

**Keywords:** heat stress, RNA-Seq, transcriptional heat responses, physiological heat responses, spikelet fertility, Brazilian flooded rice

## Abstract

High-temperature events are projected to increase in frequency under future climate scenarios, threatening rice yields globally. This study investigated the physiological and molecular responses of two Brazilian flooded rice varieties, IRGA 428 and BR-IRGA 409, during the anthesis stage under high-temperature stress, aiming to uncover mechanisms of heat tolerance. Plants were exposed to a daytime temperature of 38°C for 7 h across 3, 5, or 7 days. Prolonged heat stress led to a significant reduction in filled grain in both cultivars, although BR-IRGA 409 demonstrated greater heat tolerance, particularly under 3 days of stress, as it maintained higher spikelet fertility compared to IRGA 428. Comparative transcriptome analysis revealed that BR-IRGA 409 had more differentially expressed genes in response to heat stress, including a significant upregulation of canonical heat-responsive genes such as heat shock factors, heat shock proteins, and peptidyl-prolyl isomerase FK506-binding proteins (FKBPs). Furthermore, BR-IRGA 409 displayed enhanced modulation of the mitochondrial electron transport pathway, which is crucial for adenosine triphosphate (ATP) synthesis and cellular energy production. Interestingly, while photosynthetic performance varied between cultivars, only a few genes associated with photosynthesis were significantly altered in response to heat stress. Instead, BR-IRGA 409 displayed a higher basal expression of photosynthesis-related genes, suggesting that this pre-adaptation might mitigate heat stress impacts on photosynthesis. The ability to preserve functional photosynthetic activity is critical for sustaining the energy-intensive process required to cope with heat stress. This study highlights the difference between the varieties in their response to heat stress and identifies candidate molecular and physiological mechanisms that contribute to maintaining cellular energy homeostasis and heat tolerance in Brazilian rice, providing valuable insights for crop improvement strategies.

## Teaser

In rice, high temperatures during flowering can reduce yields. However, genotype-specific variation can be leveraged to develop heat-tolerant varieties. Brazilian rice offers an untapped resource for genotypic diversity. This study examined how two Brazilian rice varieties respond to heat stress. BR-IRGA 409 showed greater tolerance than IRGA 428, maintaining higher grain fertility and activating more heat-responsive genes. The results suggest that maintaining energy production and a strong baseline of photosynthesis-related activity may help protect rice plants from heat damage, offering useful targets for breeding heat-tolerant varieties.

## Introduction

Rice (*Oryza sativa* L.) is a primary staple food for over three billion people worldwide, playing a vital role in global food security. With the demand for rice expected to rise by 40% by 2030 (Khush 2005), addressing the challenges posed by climate change is essential for sustaining food productivity. Among these challenges, global warming is a critical threat, with projections indicating an increase in global surface air temperature by 1.4–5.8°C by the end of the twenty-first century, including significant changes in rice-growing regions such as Brazil (Krishnan et al. 2011, Intergovernmental Panel on Climate Change 2014). Rice growth is highly sensitive to temperature, with optimal daytime and night-time temperatures of approximately 28°C and 22°C, respectively (Prasad et al. 2006). Many rice-growing regions, however, experience temperatures exceeding this range, and further increases could exacerbate yield losses. Studies show a direct correlation between temperature rises and yield declines, with a 10% yield reduction for every 1°C increase in minimum temperature during the growing season (Peng et al. 2004). High-temperature events during the flowering stage, in particular, are especially detrimental, reducing spikelet fertility through impaired anther dehiscence, pollen germination, and pollen tube growth (Satake and Yoshida 1978, Jagadish et al. 2010, Zhang et al. 2018). Even brief exposure to temperature above 35°C during anthesis can significantly reduce fertility (Jagadish et al. 2007). This highlights the need to breed rice varieties that exhibit resilience to heat stress while maintaining grain yield and spikelet fertility.

Heat stress tolerance in plants is a complex trait involving physiological, biochemical, and molecular responses. Photosynthesis is one of the most heat-sensitive processes, with heat stress causing damage to thylakoid membranes, reduced chlorophyll content, stomatal closure, Rubisco deactivation, and Photosystem II impairment (Salvucci and Crafts-Brandner 2004, Murata et al. 2007, Hasanuzzaman et al. 2013). These disruptions result in reduced energy availability and increased reactive oxygen species (ROS) production, which exacerbates cellular damage. Heat-tolerant varieties, such as Nagina22 (N22) have been shown to mitigate these effects by maintaining higher antioxidant enzyme activities, net photosynthetic rates, and transpiration under heat stress condition (Jagadish et al. 2008, Li et al. 2015, Sailaja et al. 2015).

At the molecular level, heat stress induces the expression of heat shock factors (HSFs) and heat shock proteins (HSPs), which form the first line of defence by stabilizing the protein structures and facilitating repair mechanisms (Liu et al. 2009, Chandel et al. 2013, Ohama et al. 2017, Malumpong et al. 2019). Heat-tolerant rice cultivars typically exhibit higher and sustained expression of these genes, contributing to their resilience. For example. N22 shows upregulated expression of HSFs such as *OsHsfA2a*, *OsHsfA2e*, and *OsHsfA7* during heat stress at anthesis, resulting in sustained spikelet fertility and grain yield (Sailaja et al. 2015).

Brazil, the largest rice producer outside Asia, relies heavily on the state of Rio Grande do Sul (RS), which accounts for the majority of Brazilian rice production (Coltro et al. 2017). With an average yield of 7.9 t ha^−1^, RS surpasses national and global average yields of 5.0 and 4.4 t ha^−1^, respectively (Coltro et al. 2017). However, the region faces an increasing risk of heat stress during global warming. While research in Brazil has primarily focused on improving cold tolerance in rice to address early-season challenges (Cruz et al. 2006, Cruz et al. 2013, de Freitas et al. 2019, Moraes de Freitas et al. 2019) heat waves are an increasing concern. For example, the 2014 heat wave, with temperatures reaching around 40°C during the flowering stage caused a significant yield reduction and highlights the urgency of addressing heat stress (IRGA 2014).

Despite advances in understanding heat tolerance mechanisms in Asian rice cultivars (Bheemanahalli et al. 2017, Wang et al. 2019, Wu et al. 2021, Xu et al. 2021, Ishimaru et al. 2022), studies on Brazilian varieties remain limited (Brito et al. 2016, Oliveira et al. 2019, Piveta et al. 2020). To address this gap, we investigated the physiological and molecular responses of two Brazilian cultivars, IRGA 428 and BR-IRGA 409, to high-temperature stress during the anthesis stage. By assessing growth performance, spikelet fertility, and transcriptomic changes, our study aimed to uncover key mechanisms underlying heat tolerance in these cultivars. Our study provided insights for developing heat-tolerant rice varieties suited to Brazilian rice production under future climate scenarios.

## Material and methods

### Plant material and heat stress treatment

In this study, two flooded rice cultivars (*Oryza indica* spp.) from the Germplasm Bank of Instituto Rio Grandense do Arroz (IRGA) Breeding Programme (Cachoeirinha, RS, Brazil) were selected based on their heat tolerance at reproductive stage from the preliminary experiment and their similarity in maturity and flowering time (Correia 2018). BR-IRGA 409, one of the first modern Brazilian cultivars, is known for high yield and gain quality, and IRGA 428 has a herbicide resistance gene, conferring resistance to the chemical group of imidazolinones. The temperature of 38°C at the anthesis stage (R_4_ stage, according to Counce et al. 2000) was chosen to differentiate cultivars’ sensitivity according to Jagadish et al. (2010). The experiment was conducted in a randomized experimental design, with the treatments arranged in a 2 × 3 two-factor scheme. Factor A was cultivars, and Factor B was the duration of heat stress. The number of biological replicates was ten plants per heat treatment and cultivar.

Plants were grown in plastic pots with a capacity of 400 ml containing a commercial potting soil mix (Humossolo™) and maintained under flooded conditions for the whole growth period. Plants were maintained in the greenhouse until panicle emission was observed. Upon panicle emission, plants were moved to the growth chamber (Conviron BDW40 with 600 µmol m^−2^ s^−1^, 14/10 h of day/night cycles, and 60% relative humidity) with normal (29°C) and high temperature (38°C) for 7 h during a period of 3, 6, and 7 days at the anthesis stage (Supplementary Fig. S1). After the treatment, the plants were returned to the greenhouse until harvest.

### Filled grain counting and panicle and spikelet fertility test

For the spikelet fertility test, tillers from all the plants were removed, leaving only the main stem to produce uniform main culms while growing in the greenhouse. The filled and unfilled spikelets were separated from the panicle and counted at harvest. Whether the spikelet was filled or not was determined by pressing each floret between the forefinger and the thumb. Spikelets were considered filled for both completely and partially filled grains. Spikelet fertility and the percentage of reduction in spikelet fertility were calculated (Supplementary Fig. S1) with five biological replicates.

### Gas exchange and chlorophyll fluorescence measurement

To evaluate the effect of heat stress duration on photosynthesis, photosynthetic characteristics were measured in both cultivars at anthesis stage. Net photosynthetic rate (*P*_N_), stomatal conductance (*g*_s_), intercellular CO_2_ concentration, and leaf temperature were measured on the flag leaf using LI6400XT portable photosynthesis measuring system (LI-COR Environmental, USA). Measurements were made at photosynthetically active radiation of 1000 μmol m^−2 ^s^−1^ with CO_2_ concentration maintained at 400 μmol mol^−1^ and 60% of relative humidity. The parameters were measured at the same time of the day (1100 a.m.) and in five biological replicates. Electron transport rate (ETR) was measured on flag leaf on the third, fifth, and seventh day of stress duration with a portable fluorometer (OS1-FL Chlorophyll Fluorometer, Opti-Sciences, USA). All plants were measured at the same time of the day (1100 a.m.) and in five biological replicates.

### Hydrogen peroxide content measurement

The flag leaf of the two cultivars was collected on days 3, 5, and 7 at anthesis stage. One gram of leaf tissue was first ground in liquid nitrogen with pre-chilled mortar and pestle. The ground tissue was homogenized in 0.1 mM phosphate buffer (pH 7.0) containing 0.5 M EDTA. The homogenized tissue was centrifuged at 3000 rcf for 15 min at 4°C, and the supernatant was collected. The amount of H_2_O_2_ released by the leaf was determined by the Amplex® Red reagent (Sigma-Aldrich, USA) oxidation method as described previously (Smith et al. 2004). After preparation of all solutions, an aliquot of the stock solution of Bradford reagent (Sigma-Aldrich, USA) was diluted to 5×. Individual wells of an Elisa microplate contained 195 µl of Bradford reagent, 1 µl of Amplex® Red reagent, 1 µl of extraction buffer, 1 µl of 2 µM horseradish peroxidase (Sigma-Aldrich, USA), and 5 μl of sample. BSA was used to make a standard curve. Samples were incubated for 10 min at room temperature, and emission at 587 nm after excitation at 563 nm was measured using a SpectraMax M4 spectrophotometer (Molecular Devices, USA). H_2_O_2_ content in the heat stress samples was normalized to the content in the control samples.

### Statistical analysis for physiological traits

The physiological trait data were analysed by analysis of variance using the statistical computer program SisVar version 5.6. The differences among cultivars and duration of heat treatments were estimated using the Tukey test (*P*-value <.05).

### RNA-Sequencing analysis

#### RNA extraction and RNA-Seq library preparation

Flag leaves were collected from both cultivars after 3 days of heat stress and flash-frozen in liquid nitrogen before being stored at −80°C until needed. Panicle samples were first ground in liquid nitrogen with a pre-chilled mortar and pestle. Total RNA was extracted from spikelets from the middle third of the panicle with Concert™ Plant RNA Reagent (Invitrogen, USA), according to the manufacturer’s protocol. The RNA concentration was measured with NanoDrop 2000 (Thermo Fisher Scientific, USA). Two micrograms of RNA were used for library preparation with NEBNext Poly(A) Magnetic mRNA isolation kit (New England Biolabs, USA) and NEBNext Ultra Directional RNA Library Prep Kit for Illumina (New England Biolabs, USA) according to the manufacturer’s instruction. In brief, Oligo(dT) attached to magnetic beads isolated mRNA by attaching to poly(A) modified mRNA, and then mRNA was heated to 95°C for the recommended 15 min to achieve 150–200 bp fragment sizes. After cDNA synthesis, PCR library enrichment was done using 15 cycles. Libraries were run on 2100 Agilent Bioanalyzer for quantification. Finally, libraries were diluted to 10 nmol/µl concentrations for sequencing. Next-generation sequencing was performed by the NC State University Genomic Sciences Laboratory (Raleigh, NC, USA) using Illumina Hiseq 2500 with 150-bp single-end reads. RNA-Sequencing (RNA-Seq) data are available on the Sequence Read Archive (SRA) with the project number PRJNA488163.

#### Data alignment and quantification

Initial quality control was performed using FastQC version 0.12.1 (Andrews 2010). Fastq files were trimmed and filtered low-quality reads by Trimmomatic version 0.39 (Bolger et al. 2014). Trimmed reads were aligned to the different rice reference genomes using HISAT2 version 2.2.1 (Kim et al. 2015). The library type was set to ‘firststrand’, and the rest of the parameters were kept as the default. Seven rice reference genomes used for testing alignment were listed in Supplementary Table S1 (Kawahara et al. 2013, Jain et al. 2019, Choi et al. 2020, Zhou et al. 2020, Song et al. 2021). Samtools version 1.21 (Danecek et al. 2021) was used to sort and index the bam files after alignment. HTseq version 2.0.5 (Anders et al. 2014) were used to obtain read counts per gene using the following options, -f bam, -s reverse, and -m intersection-nonempty.

#### Differential expression analysis

The raw count matrix was used for differential expression analysis with EdgeR version 4.2.0 (Robinson et al. 2010) in R version 4.4.2. Lowly expressed genes were filtered out using *filterByExpr()* in EdgeR, and TMM normalization was performed prior to dispersion estimation and genewise negative binomial generalized linear model fitting. Batch effect was included in the model to account for differences between sample batches (∼batch + conditions). Differentially expressed genes (DEGs) were identified as the genes with the log_2_ fold change cut-off of >0.5 and Benjamini–Hochberg adjusted *P*-value (false discovery rate) at <0.05. Hierarchical clustering was performed to summarize the gene expression profiles, and an eigengene, a single vector representing the overall expression pattern of each cluster across all samples, was computed using a function in the R package WGCNA (Langfelder and Horvath 2008).

#### Gene ontology enrichment analysis and overrepresentation test

Gene ontology (GO) enrichment analysis was conducted using the R package topGO (Alexa and Rahnenfuhrer 2022) with MH63 DEGs as the query set and all genes in the dataset as the reference. A comprehensive list of MH63 genes and their associated GO terms was obtained from the Rice Gene Index (RGI) database (Yu et al. 2023). GO terms with a *P*-value <0.05, identified through the weight01 algorithm with Fisher’s exact test in topGO, were considered significantly enriched. Results were visualized using the R package ggplot2.

To compare DEGs and enriched GO terms between Brazilian rice cultivars and the heat-tolerant N22 cultivar (González-Schain et al. 2016), Brazilian rice gene IDs in MH63 format were converted to N22 gene IDs in the MSU format. This conversion was performed using the MH63-to-MSU ID conversion list downloaded from RGI database (Yu et al. 2023).

For the identification of overrepresented TF families in Brazilian rice, the Brazilian rice gene IDs were converted to MSU gene IDs for compatibility with the rice TF list obtained from the PlantRegMap Transcription Factor Database (Tian et al. 2020). Overrepresentation testing was performed using the ‘fisher.test’ function in R version 4.4.2. TF families with a *P*-value <.05 were classified as over-represented.

## Results

### IRGA 428 was more heat sensitive than BR-IRGA 409

**Figure 1.**
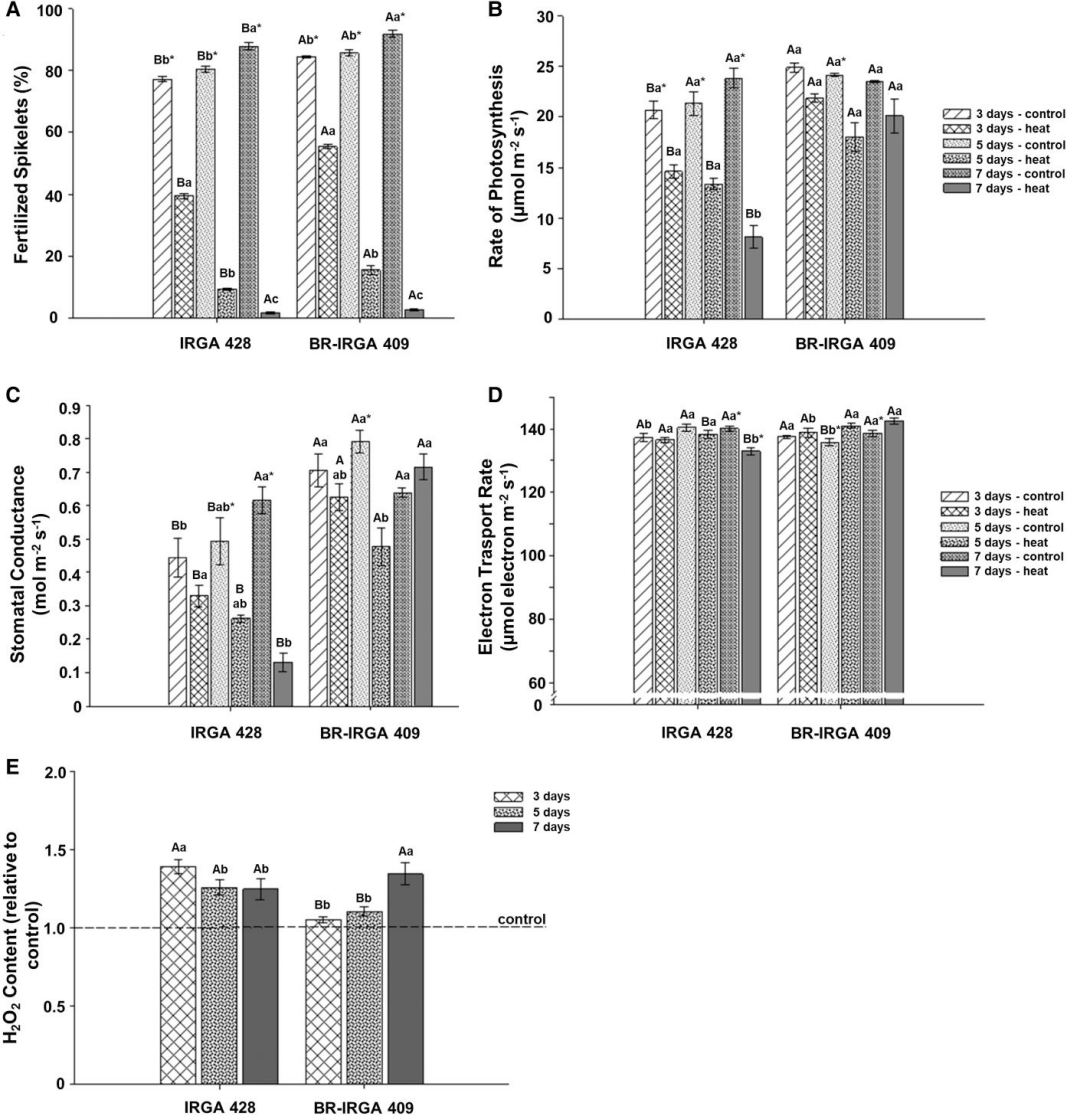
BR-IRGA 409 showed higher fertilized spikelets and better photosynthetic performance under heat stress. (a) Percentage of fertilized spikelets, (b) net photosynthetic rate, (c) stomatal conductance, (d) Electron transport rate and (e) H_2_O_2_ level under heat stress for 3, 5, and 7 days in IRGA 428 and BR-IRGA 409. Each value represents the mean of five replications ± SD. Means followed by the same capital letter indicates no significant difference among cultivars in each stress duration, and the same lowercase letters means no significant difference among stress duration in each cultivar by Tukey’s test (*P*-value <.05). Means followed by an asterisk are significantly different between control and heat stress conditions in each cultivar by Tukey’s test (*P*-value <.05).

Heat stress has a pronounced impact on spikelet fertility, making this phenotype a valuable indicator for screening heat stress tolerance in rice (Prasad et al. 2006, Cheabu et al. 2019). To assess the heat response of two Brazilian rice cultivars, plants at the anthesis stage underwent heat stress treatments for 3, 5, and 7 days. High-temperature stress negatively affected spikelet fertility in both cultivars (Fig. 1a and Supplementary Fig. S2a). As the duration of heat stress increased, spikelet fertility decreased, with the percentage of fertilized spikelets falling below 5% after 7 days of heat stress (Fig. 1a and Supplementary Fig. S2a). Notably, BR-IRGA 409 maintained significantly higher spikelet fertility than IRGA 428 after 3 and 5 days of heat stress (Fig. 1a).

Photosynthesis is one of the most susceptible processes to high-temperature stress, and Photosystem II is one of the major heat-sensitive sites in the photosynthetic apparatus (Allakhverdiev et al. 2008, Yin et al. 2010). A reduction in the net photosynthetic rate (*P*_N_) was observed across all durations of heat stress compared to the control condition (Fig. 1b). IRGA 428 exhibited a significant decrease in *P*_N_ throughout all three periods of heat stress, whereas BR-IRGA 409 showed a slight reduction under heat stress conditions (Fig. 1b). After 7 days of high-temperature stress, BR-IRGA 409 showed a maximum *P*_N_ reduction of only 14%, while IRGA 428 demonstrated a substantial 66% reduction (Fig. 1b). Stomatal conductance (*g*_s_) in IRGA 428 decreased with increasing periods of heat stress (Fig. 1c). In contrast, BR-IRGA 409 consistently maintained higher *g*_s_ than IRGA 428 at every duration of heat stress (Fig. 1b). Interestingly, in BR-IRGA 409, intercellular CO_2_ concentration (*C*_i_) remained fairly steady across the 7 days, with a slight decrease at day 5, but no significant difference at day 7 (Supplementary Fig. S2b). In contrast, *C*_i_ in IRGA 428 significantly decreased after 7 days of heat stress. In addition, heat stress duration significantly affected the ETR (Fig. 1d). IRGA 428 showed a marginal reduction in ETR, with the most significant reduction at 7 days of heat stress. However, BR-IRGA 409 showed a slight increase in ETR across all stress durations (Fig. 1d).

This contrasting trend between the two cultivars was also observed in H_2_O_2_ content (Fig. 1e). Increased antioxidant activity is a general response to heat stress (Wahid et al. 2007). H_2_O_2_ is a common signal molecule inducing cellular stress (Neill et al. 2002). IRGA 428 produced the highest H_2_O_2_ content on day 3 of heat stress, and the level decreased over time. However, BR-IRGA 409 showed an accumulation of H_2_O_2_ content over the heat stress period (Fig. 1e). Overall, BR-IRGA 409 performed better than IRGA 428 under heat stress because it showed a smaller reduction of spikelet fertility and maintained a higher photosynthetic rate.

### Transcriptome analysis reveals a contrast biological pathway in response to heat stress between two Brazilian cultivars

RNA-Seq was conducted to investigate the molecular mechanisms underlying the differential physiological performances observed between the two Brazilian rice cultivars under heat stress. Flag leaf samples were collected at 3 days under heat stress because this time point marked the maximum difference in spikelet fertility between control and heat stress conditions for both cultivars (Fig. 1a). Given that BR-IRGA 409 and IRGA 428 possess a mixture of *japonica* and *indica* backgrounds, we first determined a proper reference genome for downstream analysis. The RNA-Seq reads were aligned to seven reference genomes from four rice subpopulations (Agrama et al. 2010, Wang et al. 2018) (Supplementary Fig. S3a). The reference genomes of *O. sativa* spp. *japonica* were Nipponbare (Kawahara et al. 2013) and Kitaake (Jain et al. 2019) while MH63, ZS97 (Song et al. 2021), and IR64 (Zhou et al. 2020) were spp. *indica*. N22 (Zhou et al. 2020) and Basmati 334 (Choi et al. 2020) represented circum-Aus and circum-Basmati groups, respectively. The overall percentage of mapped reads were over 80% in all reference genomes, and there was no significant difference in the overall percentage of mapped reads between BR-IRGA 409 and IRGA 428 in Nipponbare, MH63, ZS97, and N22, and Basmati 334 (Supplementary Fig. S3a). Interestingly, Brazilian rice showed a high percent uniquely mapped reads to the *indica* reference genomes (MH63, ZS97, and IR64) compared to other rice subpopulations, suggesting that the sequences of Brazilian varieties were more similar to *indica* subspecies than *japonica* subspecies.

Since the choice of reference genome can significantly impact downstream analysis (Slabaugh et al. 2019), we performed differential expression analysis using gene counts from seven reference genomes. We first assessed sample associations using PCA plots (Supplementary Fig. S3b), which showed similar clustering patterns regardless of the reference genome used. Within the BR-IRGA 409 variety, control and heat-stressed samples were more distinct from each other than those in IRGA 428, suggesting that gene expression in IRGA 428 was less altered after 3 days of heat stress. Additionally, PCA indicated greater dispersion among biological replicates in IRGA 428, likely due to the experimental design, where replicates were collected from three independent experiments. This variation between experiments could influence downstream analysis, so we accounted for batch effects in the differential expression analysis.

Differential expression analysis revealed that IRGA 428 had fewer DEGs than BR-IRGA 409 across all reference genomes (Supplementary Fig. S3c), further supporting the PCA results that IRGA 428 exhibited minimal gene expression changes after 3 days of heat stress. While IRGA 428 showed more downregulated genes under heat stress, BR-IRGA 409 had a higher number of upregulated genes (Supplementary Fig. S3c). As homologous genes between Nipponbare and each of MH63, N22, ZS97, and IR64 had already been identified (Yu et al. 2023), 65% of all detected genes were found to be homologous across the five reference genomes (Supplementary Fig. S4a). In IRGA 428 samples, approximately 47% of heat-downregulated genes and 34% of heat-upregulated genes were shared among five reference genomes (Supplementary Fig. S4b and c). The remaining DEGs overlapped with one to four reference genomes, with less than 10% and 15% for downregulated and upregulated genes respectively. About 7% of downregulated and 14% of upregulated genes in IRGA 428 were unique to the Nipponbare genome. A similar pattern was observed in BR-IRGA 409, where 45% of downregulated and 48% of upregulated genes were shared across all reference genomes (Supplementary Fig. S4d and e). Nipponbare also showed the highest percentage of unique DEGs compared to other reference genomes, even though the percentage was not outstanding from the others. Given that the MH63 reference genome yielded the highest overall mapped reads, we used its results for downstream analysis.

**Figure 2.**
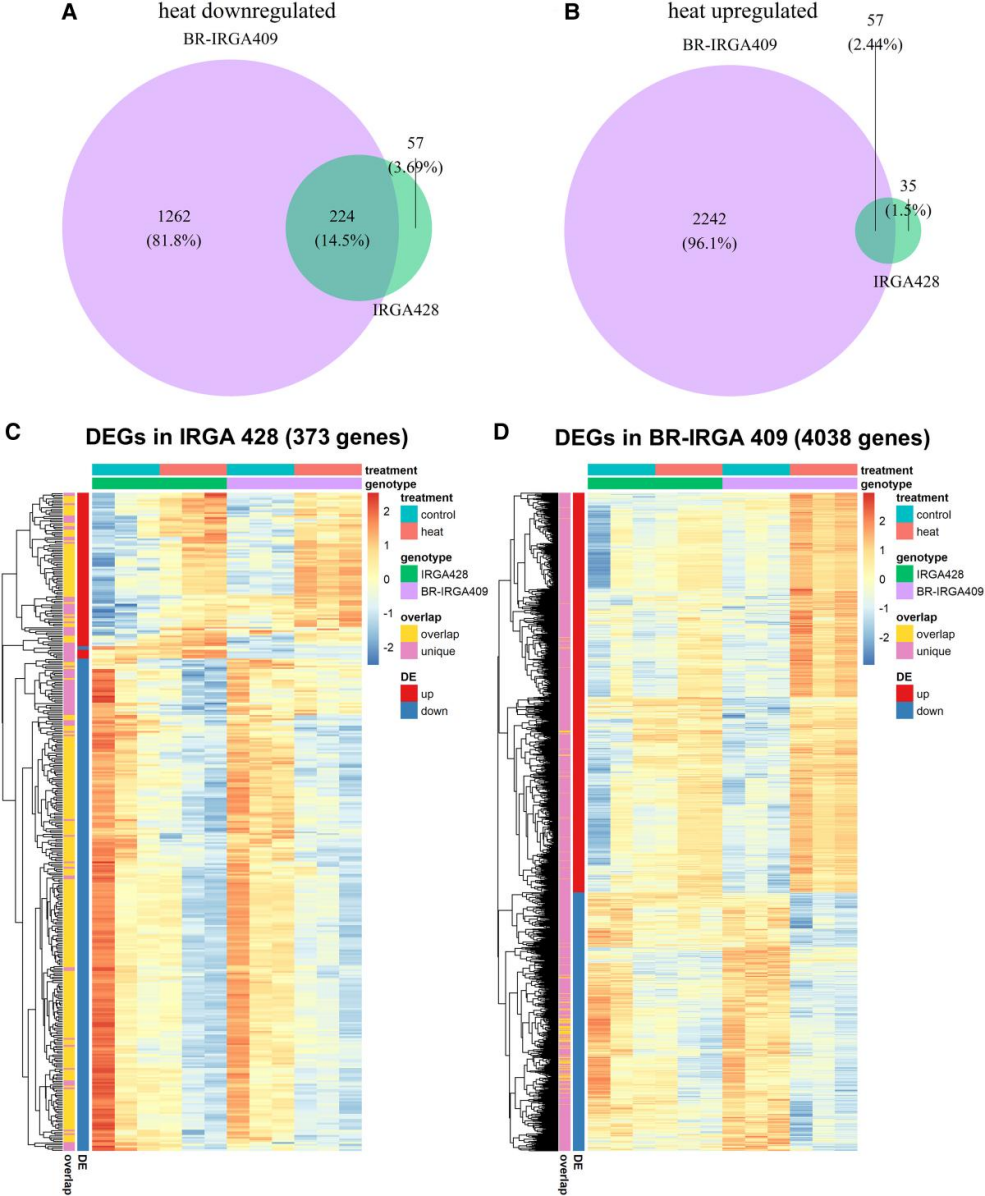
Heat stress affected gene expression in Brazilian rice cultivars. (a and b) Venn diagrams showing the number of heat downregulated (a) and upregulated genes (b) in IRGA 428 and BR-IRGA 409. (c and d) Heatmaps showing log_2_CPM of DEGs identified in IRGA 428 (c) and BR-IRGA 409 (d).

Venn diagrams comparing DEGs identified using the MH63 reference genome showed the overlap between IRGA 428 and BR-IRGA 409 in both heat downregulated (224 genes) and upregulated (57 genes) categories (Fig. 2 and Supplementary Tables S2 and S3). While most of the heat-responsive DEGs in IRGA 428 are shared with BR-IRGA 409, most of the DEGs in the heat tolerant variety, BR-IRGA 409 are not identified as DEGs in IRGA 428. This suggests that heat stress induced distinct gene expression responses in BR-IRGA 409.

**Figure 3.**
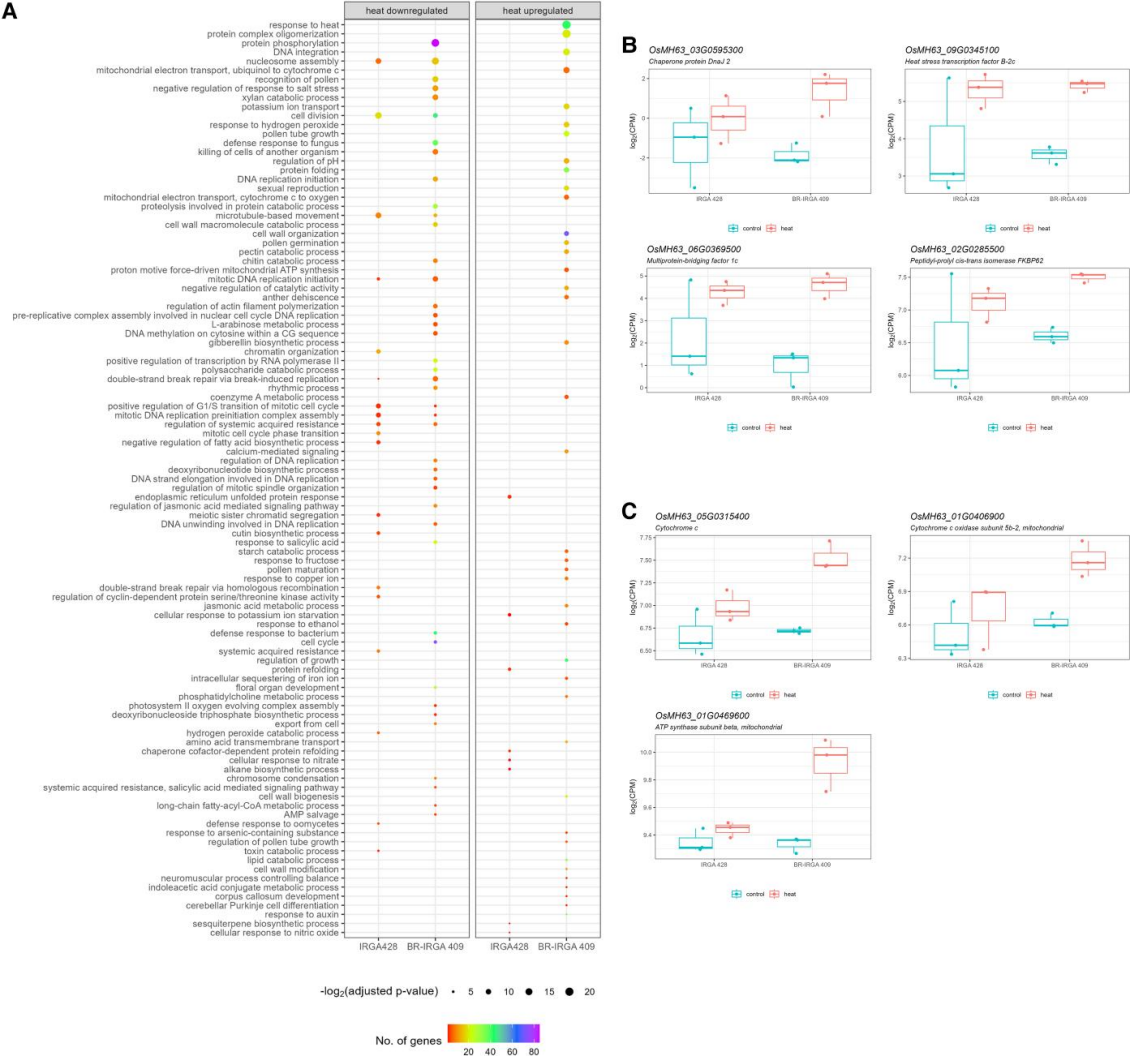
Two Brazilian rice displayed distinct biological processes in response to heat stress. (a and b) Dot plots of significant GO terms (adjusted *P*-value <.05) of heat downregulated (a) and upregulated (b) genes in IRGA 428 and BR-IRGA 409. (b and c) Examples of heat upregulated genes in BR-IRGA 409 that were present in the ‘response to heat’ GO term (b) and GO terms related to the mitochondrial electron transport (c).

To further explore the biological functions underlying these differences, we performed GO enrichment analysis. As more DEGs were identified in BR-IRGA 409, there were more significant GO terms identified in this heat-tolerant variety compared to IRGA 428, including many that are unique to BR-IRGA 409 (Fig. 3a). In both cultivars, heat downregulated genes were enriched in pathways related to cell division, such as cell division and nucleosome assembly (Fig. 3a and Supplementary Fig. S5a). Examples of genes associated with the cell division GO term include *OsMH63_03G0401800* (*Cyclin A3-1*) and *OsMH63_06G0484000* (*Cyclin-B2-2*). Similarly, genes involved in nucleosome assembly include *OsMH63_08G0386700* (*Histone H2B.2*) and *OsMH63_02G0454700* (*Histone H4*). Interestingly, BR-IRGA 409 displayed significant enrichment in protein phosphorylation and rhythmic process GO terms, which were not observed in IRGA 428 (Fig. 3a and Supplementary Fig. S5b). Examples of genes involved in protein phosphorylation include *OsMH63_07G0428000* (*Cysteine-rich receptor-like protein kinase 10*) and *OsMH63_04G0477300* (*wall-associated receptor kinase 5*). These transcripts show little change in IRGA 428, but are significantly downregulated in BR-IRGA 409. Several genes related to the rhythmic process including the PSEUDO-RESPONSE REGULATOR PROTEINS (PRRs) and REVEILLE (RVE) family were identified as DEGs in the heat-tolerant BR-IRGA 409, but were not identified in IRGA 428. For some rhythmic regulators including OsMH63_07G0482100 (*PRR37*), (*OsMH63_04G0465500* (*RVE2*), and *OsMH63_06G0004400* (*RVE8*)), there is significant and large reduction in transcript levels in BR-IRGA 409 (Supplementary Fig. S5b). However, there is also a downward trend in the IRG 428 samples as well, although the reduction in levels is much less than in BR-IRGA 409 and is not identified as a significant difference. The overall difference in the transcriptional levels of genes related to rhythmic processes suggests that the impact of heat stress on the circadian clock may have significant differences betweenBR-IRGA 409 and IRGA 429, and these differences could result in differences observed in circadian-related genes.

For heat upregulated genes, the most significant GO term in BR-IRGA 409 was response to heat (Fig. 3a and b). This GO term included numerous heat stress-related genes, such as *chaperone protein DnaJ* (*OsMH63_01G0665400* and *OsMH63_03G0595300*), *Class I HSP* (*OsMH63_03G0149500*), *HSFB2c* (*OsMH63_09G0345100*), *MBF1C* (*OsMH63_06G0369500*), and *FKBP62* (*OsMH63_02G0285500*). These genes showed increased transcript levels in both BR-IRGA 409 and IRGA 428, consistent with their known roles in heat stress responses. Other significant GO terms in BR-IRGA 409 were associated with the mitochondrial electron transport pathway (Fig. 3c), which plays a crucial role in heat stress tolerance by providing adenosine triphosphate (ATP) to support protein turnover—an energy-intensive process (Scheurwater et al. 2000). Key enriched GO terms included ‘ubiquinol to cytochrome *c*’ [e.g. *OsMH63_05G0315400* (*cytochrome c*)], ‘cytochrome *c* to oxygen’ [e.g. *OsMH63_01G0406900* (*cytochrome c oxidase subunit 5b-2*)], and ‘proton motive force-driven mitochondrial ATP synthesis’ [e.g. *OsMH63_01G0469600* (*ATP synthase subunit beta*)]. These transcripts show significantly higher levels in BR-IRGA 409, but are not identified as DEGs in IRGA 428. This suggests that this pathway may be more active in BR-IRGA 409 under heat stress, enhancing energy production to meet increased energy demands under heat stress while mitigating ROS production. In contrast, IRGA 428 had few significantly enriched GO terms, primarily related to the ER unfolded protein response and protein refolding (Fig. 3a and Supplementary Fig. S5c). For example, Heat shock 70 kDa proteins *BIP2* and *BIP4* (*OsMH63_03G0492800* and *OsMH63_05G0322100*, respectively) were involved in the ER unfolded protein response.

### The interaction between cultivar and heat stress shows unique gene expression in response to heat stress in Brazilian rice

**Figure 4.**
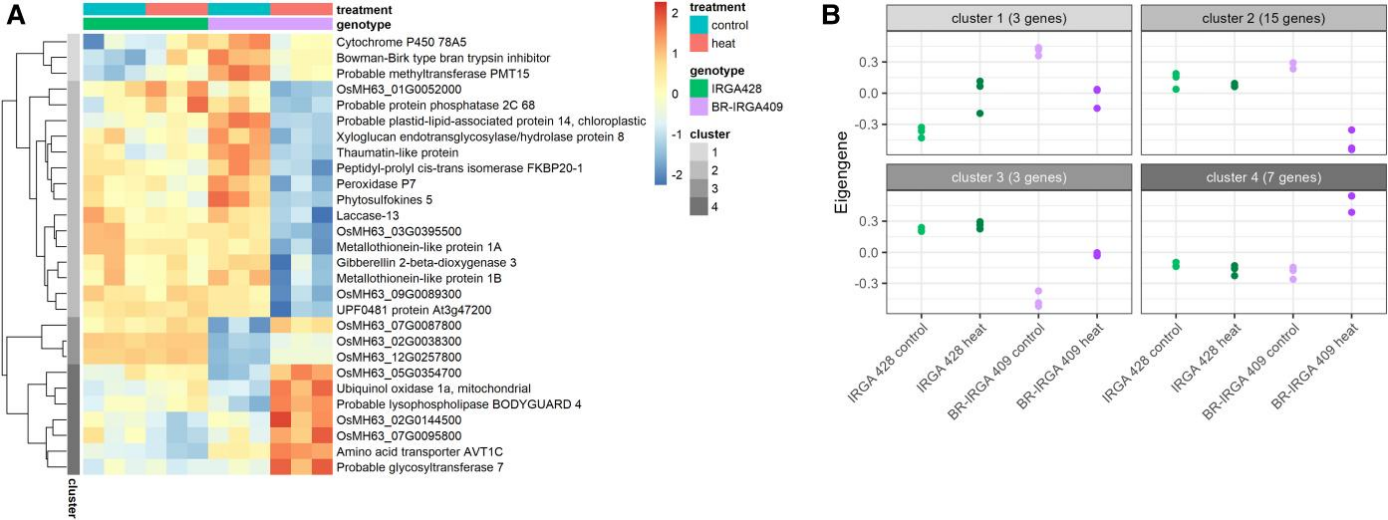
Interaction effect of rice cultivar and heat stress on gene expression in Brazilian rice. (a) Heatmaps showing log_2_CPM of DEGs derived from the interaction effect of rice cultivar and heat stress treatment. (b) Eigengenes representing gene expression profiles of DEGs from the interaction effect.

To further investigate transcriptional differences between cultivars under heat stress, we used EdgeR to identify genes affected by the interaction between heat stress and cultivar (Fig. 4a). This analysis identified only 28 DEGs, with their associated GO terms listed in Supplementary Table S4. These genes were involved in diverse biological processes and could be categorized into four clusters based on their expression patterns (Fig. 4b). Cluster 1 contained genes upregulated in IRGA 428 but downregulated in BR-IRGA 409 under heat stress. The differential response of these transcript levels is primarily due to their differences in basal levels prior to the heat stress. For example, *OsMH63_03G0393900* (*Cytochrome P450 78A5*) exhibited higher basal expression in BR-IRGA 409 than in IRGA 428. Cytochrome P450 (CYP) plays a crucial role in the biosynthesis of secondary metabolites, antioxidants, and phytohormones in higher plants, as well as in heat stress response (Kilasi et al. 2018, Pandian et al. 2020, Cai et al. 2023). Cluster 2 included genes that were significantly downregulated under heat stress only in BR-IRGA 409, such as *OsMH63_01G0599100* (*FKBP20-1*), which is involved in protein folding, and *OsMH63_06G0326900* (*Peroxidase P7*), which plays a role in oxidative stress response. Cluster 3 comprised genes with lower expression levels in BR-IRGA 409 than in IRGA 428. These genes were significantly lower in control conditions in BR-IRGA 409 compared to IRGA 428 and were upregulated in heat stress in BR-IRGA 409. All three genes in this cluster lacked known annotations or biological functions in the MH63 reference genome, but two of them (OsMH63_07G0087800 and OsMH63_12G0257800) have homologues in the Nipponbare reference genome as LOC_Os07g09814 (F-box domain containing protein) and LOC_Os12g31660 (harbinger transposase derived 1), respectively (Jiang et al. 2023, Yu et al. 2023) Cluster 4 contained seven genes upregulated under heat stress exclusively in BR-IRGA 409. Examples included *OsMH63_04G0478600* (*Ubiquinol oxidase 1a*), a mitochondrial enzyme involved in alternative respiration; *OsMH63_07G0473500* (*Lysophospholipase BODYGUARD 4*), which plays a role in cell wall organization; and *OsMH63_02G0000700* (*amino acid transporter AVT1C*), which facilitates amino acid transmembrane transport. These findings highlight distinct transcriptional responses between the two cultivars.

### Transcription factor families responding to heat stress in Brazilian varieties

**Figure 5.**
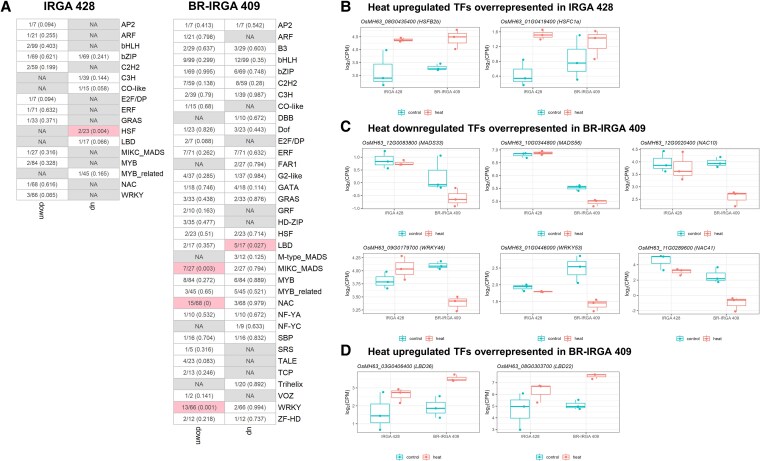
Distinct groups of TFs were overrepresented in two Brazilian rice cultivars. (a) Heatmaps showing Fisher's exact test of TFs that were downregulated and upregulated under heat stress in IRGA 428 and BR-IRGA 409. In each box, the number outside the bracket is a ratio of the number of differentially expressed TFs to the total number of TFs in the family, and the number inside the bracket is a *P*-value. The boxes highlighted in pink indicate significant overrepresentation at *P*-value <.05, and white colour means no significant overrepresentation. (b) Boxplots showing the expression of HSF genes overrepresented under heat stress in IRGA 428. (c) Boxplots showing the expression of example genes in MIKC_MAD, NAC, and WRKY families overrepresented in response to heat stress in BR-IRGA 409. (d) Boxplots of example genes in LBD family that were overrepresented under heat upregulated in BR-IRGA 409.

To understand the regulation of gene expression, we focused on transcription factor (TF) families. We identified overrepresentation TF families that are differentially expressed under heat stress between two Brazilian rice varieties using Fisher’s exact test. In the heat-susceptible line IRGA 428, no TF family was significantly overrepresented among heat downregulated genes (Fig. 5a). The HSF family was the only significantly overrepresented TF family among heat-upregulated genes in IRGA 428, comprising *OsMH63_08G0435400* (*HSFB2b*) and OsMH63_01G0419400 (*HSFC1a*) (Fig. 5b). The HSF family is known for its role in heat stress tolerance, as it regulates the expression of HSPs, which assist in protein folding and degradation during stress (Ohama et al. 2017).

In contrast, BR-IRGA 409 exhibited a distinct transcriptional response. The MIKC-type MADS, NAC, and WRKY families were overrepresented among heat downregulated genes, while the lateral organ boundaries (LBD) family was overrepresented among heat upregulated genes (Figs. 5a, c, and d). The MIKC-type MADS family [e.g. *OsMH63_12G0083800 (MADS33)* and *OsMH63_10G0344800* (*MADS56*)], which is primarily involved in regulating flower and root development, may also play a role in stress tolerance through its regulation of developmental pathways under heat stress (Castelán-Muñoz et al. 2019). NAC transcription factors [e.g. *OsMH63_12G0020400* (*NAC10*) and *OsMH63_11G0289600* (*NAC41*)] are known to be key players in plant stress responses, including heat, by regulating stress-responsive genes and mediating adaptive responses (Fujita et al. 2006). The WRKY family [e.g. *OsMH63_09G0179700* (*WRKY46*) and *OsMH63_01G0446000* (*WRKY53*)], which is involved in plant defence and stress responses, has been shown to regulate heat stress-induced gene expression through intricate signalling pathways (Cheng et al. 2021). Finally, the LBD family (*OsMH63_03G0406400* (*LBD36*) and *OsMH63_08G0303700* (*LBD22*), typically involved in regulating organ development, has not been characterized in the context of heat stress response in crops (Baillo et al. 2019). Overall, these results suggest that the two Brazilian rice varieties regulate different sets of transcription factors in response to heat stress, potentially reflecting divergent strategies for managing heat-induced damage.

## Discussion

### Physiologic response to high-temperature stress in Brazilian rice

The effect of heat stress on rice growth and development has been studied for decades. Most studies focus on Asian rice with short durations of heat stress during either the vegetative or reproductive phase (Prasad et al. 2006, Jagadish et al. 2010, Hirabayashi et al. 2015, Sailaja et al. 2015, González-Schain et al. 2016, Takai et al. 2020, Wahab et al. 2020, Zafar et al. 2022). Brazil is one of the world’s leading rice-producing countries, but research on heat stress in Brazilian rice is minimal. We determined the effect of heat stress duration on two Brazilian rice cultivars at the anthesis stage because rice is most susceptible to heat stress during the flowering period, and spikelet fertility can be used to determine the level of heat tolerance (Jagadish et al. 2010, Sailaja et al. 2015, Malumpong et al. 2019). Our study showed that increasing the duration of the heat stress significantly decreased yield as more than 95% of spikelet fertility reduction was observed at Day 7 of heat stress in both cultivars (Fig. 1a and Supplementary Fig. S2a). On Day 3 of heat stress, BR-IRGA 409 significantly retained higher spikelet fertility than IRGA 428 (Fig. 1a). Our results were consistent with the morpho-physiological characteristics of the well-known heat-tolerant rice cultivar N22, which showed less reduction in *P*_N_, increased gas exchange, and increased antioxidant enzyme activity, supporting a minimal reduction in spikelet fertility (Jagadish et al. 2010, Sailaja et al. 2015). However, the spikelet fertility in BR-IRGA 409, which was close to 60% under 38°C for 3 days, was lower than the published performance of N22. N22 maintained over 80% of spikelet fertility in experiments under 36°C for 2 h (Jagadish et al. 2008) and around 60% of spikelet fertility under conditions where the temperature was elevated 1°C–5°C from the seedling stage (Sailaja et al. 2015). We can consider BR-IRGA 409 as moderately short-term heat tolerant and IRGA 428 as heat susceptible according to previous studies classifying heat stress tolerance in rice (Jagadish et al. 2010, Sailaja et al. 2015).

Few publications report on heat stress response in Brazilian rice. Piveta et al. (2020) found that the photosynthetic rate increased in two rice varieties IRGA 424 and SCS119 Rubi under heat shock (42°C for 2 h). However, both genotypes showed a reduction in the number of tillers (20% and 10% reduction in IRGA 424 and SCS119 Rubi, respectively) and grain yield (20% reduction in both varieties) (Piveta et al. 2020). In the rice cultivar Puitá INTA-CL, applying 40°C for 28 h during the vegetative stage resulted in a reduction of photosynthetic rate and increased antioxidant enzyme activity (Oliveira et al. 2019). Brito et al. (2016) determined heat stress in three Embrapa cultivars: BRS Pampa, BRS Sinuelo CL, and IAS 12-9 Formosa under the mean maximum air temperatures reaching 36.8°C during the initial flowering phase for 2 days and found that heat stress negatively affected photosynthetic rate at 24 h across the three genotypes. However, the effect on spikelet fertility and grain yield between the varieties did not correlate with the change in photosynthesis (Brito et al. 2016). Spikelet sterility in BRS Sinuelo CL was up to 54%, while the other two varieties had around 20% of spikelet sterility (Brito et al. 2016). These studies suggest that the initial photosynthetic response to heat stress may not be a good indicator of heat tolerance as measured by spikelet fertility and yield. Here, we present a more detailed investigation into the initial physiological and transcriptional heat responses of the heat-sensitive IRGA 428 and the moderately heat-tolerant BR-IRGA 409 Brazilian cultivars. These results provide new insights into the heat stress response of two Brazilian rice cultivars that have not been studied before. Additionally, we highlight the critical importance of considering the basal, pre-stress expression levels to studies that seek to use transcriptional responses to heat stress in rice breeding programs.

We measured photosynthetic performance, H_2_O_2_ content, and transcriptional changes to understand the physiological and biochemical responses to heat stress during the reproductive phase. On Day 3 of heat stress, BR-IRGA 409 had a higher photosynthetic rate (*P*_N_), and lower H_2_O_2_ accumulation than IRGA 428, consistent with the significantly higher spikelet fertility (Fig. 1a and b). IRGA 428 showed a marginal reduction in the ETR under heat stress, but ETR slightly increased in BR-IRGA 409 over time, suggesting that this cultivar gradually adapted to high-temperature conditions. The reduction of ETR under heat stress is due to the inactivation of the oxygen-evolving complex and a decrease in the utilization of NADPH and ATP resulting from reduced photosynthesis (Havaux 1993, Luo et al. 2011). These physiological heat stress responses indicated that IRGA 428 had reduced photosynthetic activity and increased ROS production, which could potentially result in reduced spikelet fertility. On the other hand, BR-IRGA 409, which had a small reduction in spikelet fertility, maintained high photosynthesis and low ROS production.

#### Induction of canonical heat-responsive genes was observed in both Brazilian rice cultivars

High temperature is a major abiotic stress that limits plant growth and development globally. Several studies have used RNA-Seq technology to investigate the transcriptional response to heat stress in rice (Yang et al. 2015, González-Schain et al. 2016, Desai et al. 2021, Liu et al. 2020, Vitoriano and Calixto 2021). We performed RNA-Seq on flag leaves collected on Day 3 of heat stress, as this time point showed the largest difference in spikelet fertility between the two cultivars (Fig. 1a). Due to the lack of a reference genome for Brazilian rice, we aligned sequencing reads to multiple available rice reference genomes. More than 95% of reads mapped to indica reference genomes (Supplementary Fig. S3a), suggesting that these rice varieties may have significant components of the indica subspecies. Differential expression analysis across the five reference genomes revealed that fewer than 50% of DEGs were consistently identified across all genomes, while the remainder were detected in only one to four genomes (Supplementary Fig. S4). This fragmentation highlights the limitations of using non-native reference genomes and addresses the importance of developing a high-quality reference genome and gene annotation specific to Brazilian rice.

Canonical heat stress-responsive genes, including *HSPs* and *HSFs* (Ohama et al. 2017, Guihur et al. 2022) were unsurprisingly upregulated in both rice cultivars (Figs. 3b and 5b). The general response pattern of HSF transcripts is either a rapid, early response to heat stress followed by a return to the pre-stress state, or a constitutive upregulation throughout the heat treatment (Ohama et al. 2017, Guihur et al. 2022). Not all *HSFs* induced by high temperatures maintain their expression throughout prolonged stress (Wang et al. 2020). Therefore, these genes may not be reliable indicators of heat tolerance beyond the initial 1–2 h after heat stress exposure. Consistent with this, we observe that some classic heat shock response genes, such as *OsMH63_03G0595300*, a gene encoding a DnaJ chaperon protein, remained highly induced in response to heat stress in the heat-tolerant BR-IRGA 409 but were less induced in the heat-susceptible IRGA 428 genotype (Fig. 3b). Furthermore, it has been reported that the transcriptional regulation of these basal heat responses differs based on the cultivar, temperature degrees, the duration of heat treatment, and the developmental stage in which the stress is applied (Zhang et al. 2012, González-Schain et al. 2016).

The interaction effect of heat stress and cultivar revealed distinct gene expression patterns between BR-IRGA 409 and IRGA 428, despite identifying only a small number of DEGs (Fig. 4). For example, CYP 78A5 gene responded differently to heat stress in the two cultivars: it was upregulated in IRGA 428 but downregulated in BR-IRGA 409. CYP plays a role in heat stress response, and QTL analysis of root and shoot growth between N22 and IR64 identified CYP genes as potential candidates for heat tolerance (Kilasi et al. 2018). A study of two indica varieties found an upregulation of CYP genes in the heat-tolerant variety under heat stress (Cai et al. 2023). Interestingly, despite BR-IRGA 409 being heat-tolerant, it showed downregulation of *CYP 78A5* gene under heat stress, although it exhibited higher basal expression under the normal condition compared to IRGA 428. This suggests that a higher baseline level of *cytochrome P450* gene may enable BR-IRGA 409 to better cope with heat stress.

As no high-throughput transcriptional analysis of heat stress has been conducted in Brazilian rice, we compared DEGs under heat stress in IRGA 428 and BR-IRGA 409 to the gene expression profile of the heat-tolerant cultivar N22 (González-Schain et al. 2016) to investigate similarities and differences in their heat stress responses (Supplementary Fig. S6 and Supplementary Tables S5 and S6). The Venn diagrams showed only a few overlapping genes between three rice cultivars (15 downregulated and one upregulated gene). However, there are more overlapping DEGs between N22 and the heat tolerant line BR-IRGA 409 than between heat-sensitive IRGA 428 and N22 (Supplementary Fig. S6a). Examining GO enrichment of the heat responsive genes in each cultivar indicated that the genes downregulated by heat were involved in various pathways (Supplementary Fig. S6b). In contrast, the heat upregulated genes showed enrichment of similar pathways, especially heat response and protein folding. Most of the heat upregulated genes shared between BR-IRGA 409 and N22 were HSPs (Supplementary Table S6), suggesting that these cultivars possibly shared a canonical heat stress response pathway.

Another interesting overlapping gene was *OsFKBP62b* (*LOC_Os02g28980*), which was induced by heat stress in both BR-IRGA 409 and N22 (Supplementary Table S6g). *OsFKBP62b* is a close homologue of Arabidopsis *ROF1* (*AtFKBP62*), a member of the FK506 binding protein (FKBP) family that plays a key role in thermomemory (Charng et al. 2007, Meiri and Breiman 2009, Lämke et al. 2016, Thirumalaikumar et al. 2021). ROF1 interacts with HSP90.1, and this complex is translocated to the nucleus during heat stress, where HSP90.1 interacts with HSFA2, a key regulator of acquired thermotolerance, to induce the expression of HSFA2-regulated *HSPs* (Meiri and Breiman 2009, Thirumalaikumar et al. 2021). González-Schain’s study noted that *OsFKBP62b* was highly expressed in the heat-susceptible cultivar IR64 under normal conditions and slightly induced by heat stress, whereas it was strongly induced by heat stress in the heat-tolerant cultivar N22 (González-Schain et al. 2016). Our study found that *OsFKBP62b* was significantly upregulated only in BR-IRGA 409 under heat stress, suggesting that this might be a key regulator giving the different physiological response between the two Brazilian cultivars (Supplementary Fig. S6g).

There were a large number of non-overlapping genes between Brazilian rice and N22, which could be due to differences in experimental setup or distinct molecular mechanisms in response to heat stress. For example, *HSFA2a* (*LOC_Os03g53340*), a canonical HSF, was strongly induced in N22 in response to heat stress (González-Schain et al. 2016), but it was not induced in either Brazilian cultivar. The differences between N22 and the two Brazilian cultivars, particularly the heat-tolerant BR-IRGA 409, suggested that the Brazilian cultivars may have unique mechanisms of heat tolerance not found in N22. Critically, the majority of the canonical heat-responsive genes show a consistent upregulation in both BR-IRGA 409 and N22, this suggests that the observed physiological differences may not be due to differences in the classic heat response pathway. One interpretation of these results was that Brazilian rice might offer valuable resources for improving heat tolerance in rice. If the heat tolerance mechanisms of N22 are, in fact, distinct from those of BR-IRGA 409, combining these mechanisms could enhance heat tolerance in Brazilian rice and potentially other rice varieties.

#### Differentially expressed genes between the sensitive and tolerant cultivars include mitochondrial electron transport chain-associated genes

We observed that BR-IRGA 409 maintained a higher photosynthetic rate than IRGA 428. However, differential expression analysis from RNA-Seq revealed minimal differences in the expression of photosynthesis-related genes in response to heat stress between the two cultivars (Supplementary Fig. S7). Among the 111 photosynthesis-related genes identified in the RNA-Seq dataset, only two genes in IRGA 428 and seven genes in BR-IRGA 409 were classified as differentially expressed (Supplementary Fig. S7). Clustering the photosynthesis-related genes into four groups based on expression patterns revealed that Cluster 2 was the only group showing no difference in basal expression between IRGA 428 and BR-IRGA 409 under control conditions (Supplementary Fig. S7). In contrast, the other three clusters exhibited higher expression in BR-IRGA 409 compared to IRGA 428 under control conditions. This elevated basal expression in BR-IRGA 409 could potentially contribute to its enhanced performance under heat stress.

Conversely, BR-IRGA 409 demonstrated a significant increase in the expression of genes related to the mitochondrial electron transport chain, including those encoding proteins involved in cytochrome c, cytochrome oxidase, and ATP synthase (Fig. 3a and c). This suggests that the high expression of these genes may help maintain ATP production in the cells, which is crucial for the response to heat stress. Our physiological and RNA-Seq data indicated that the increased heat tolerance of BR-IRGA 409 may be associated with enhanced basal expression of photosynthesis-related genes and sustained high expression of mitochondrial electron transport genes. Since heat tolerance is energy-intensive, the ability to maintain basal metabolic processes likely contributes to a higher tolerance and more successful recovery.

## Conclusion

Heat stress significantly impacted spikelet fertility and photosynthesis in Brazilian flooded rice. BR-IRGA 409 demonstrated the ability to maintain more efficient photosynthesis and higher spikelet fertility than IRGA 428, highlighting the genetic variability in these Brazilian rice cultivars that resulted in distinct responses to heat stress. Comparative transcriptome analysis revealed a set of DEGs that responded to heat stress in both cultivars, including canonical heat-responsive genes, as well as unique sets of DEGs specific to each cultivar indicating that genetic variability in these Brazilian rice cultivars led to differences in their response to heat stress. Comparative transcriptome analysis revealed a set of DEGs that responded to heat stress in both cultivars, including canonical heat-responsive genes, as well as a unique set of DEGs in each cultivar. Notably, BR-IRGA 409 exhibited sustained high expression of genes associated with energy production, particularly those involved in the mitochondrial electron transport chain, which may help maintain ATP production under heat stress. Our results suggested that the heat tolerance of BR-IRGA 409 is possibly an energy-intensive process, with the ability to preserve photosynthetic capacity likely linked to its enhanced basal metabolic processes and efficient energy management during heat stress.

## Supplementary Material

plaf043_Supplementary_Data

## Data Availability

RNA-Seq data are available on the NCBI Sequence Read Archive (SRA) with the project number PRJNA488163.
