# Supplementary material for: Physiological and molecular responses to high-temperature stress at anthesis in Brazilian flooded rice
Source: AoB Plants. 2025 Aug 23;17(5):plaf043. doi: 10.1093/aobpla/plaf043 (PMC12449062; doi:10.1093/aobpla/plaf043)
Supplement: plaf043_Supplementary_Data [file plaf043_supplementary_data.zip › Supplemental Figs.pdf]

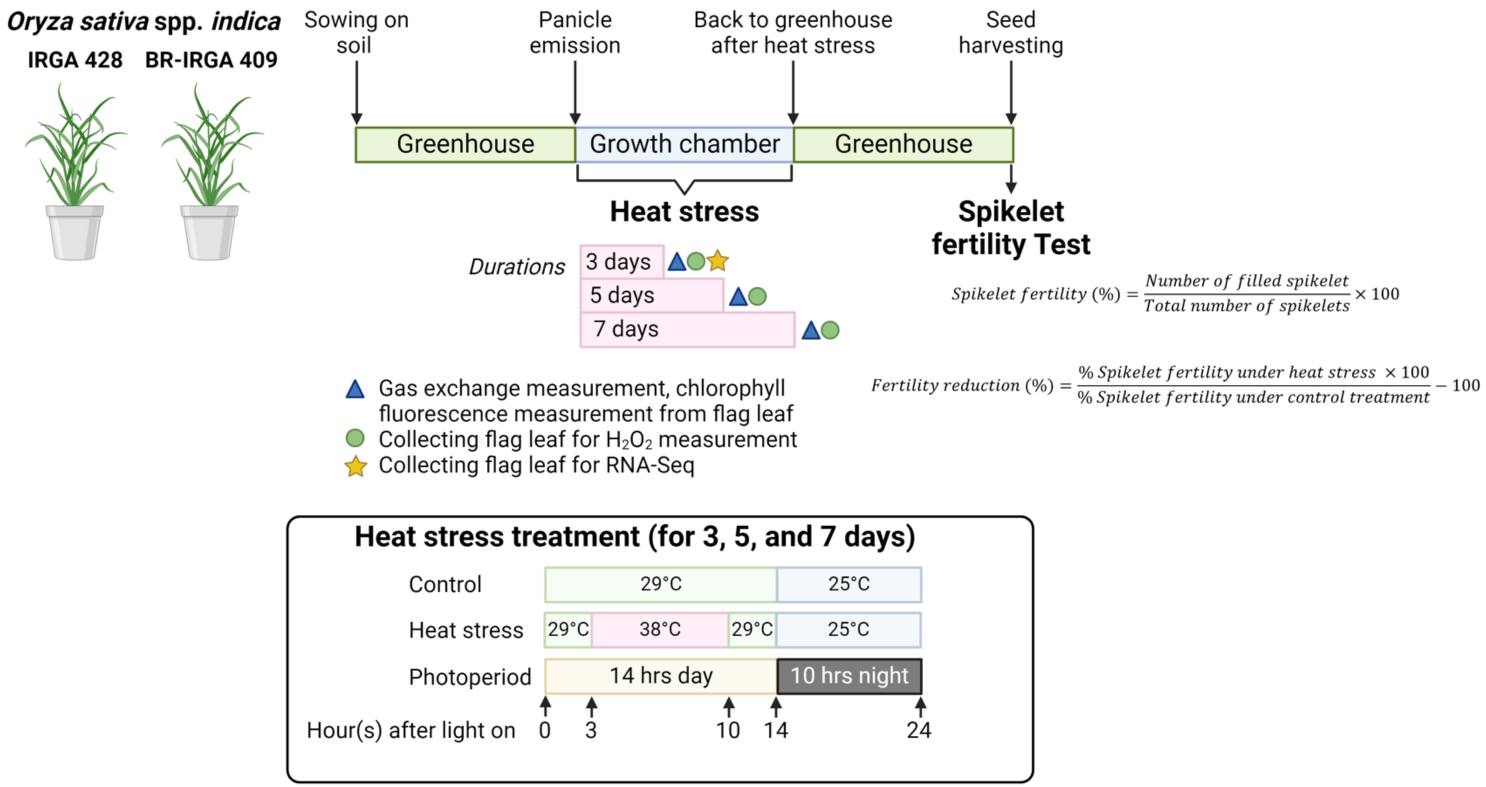

Supplemental Figure 1. Heat stress experiment design. See Materials and Methods for details.

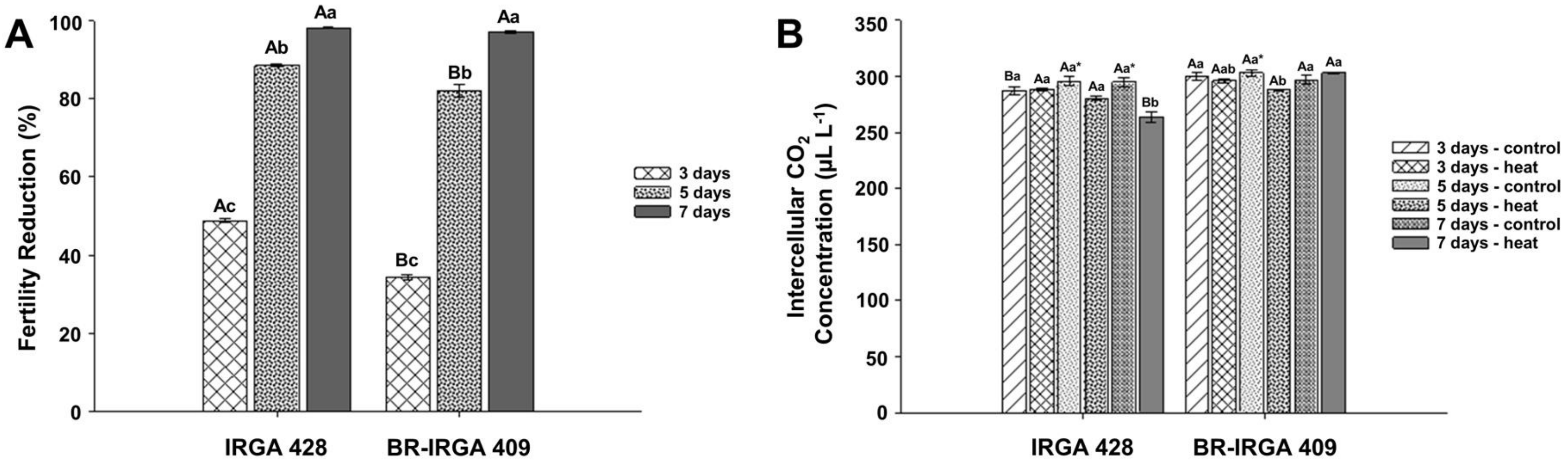

Supplemental Figure 2. (A) The percentage of fertility reduction and (B) intercellular CO_2 concentration under heat stress for three, five, and seven days in IRGA 428 and BR-IRGA 409. Each value represents the mean of five replications ± SD. Means followed by the same capital letter indicate no significant difference among cultivars in each stress duration, and the same lowercase letters mean no significant difference among stress duration in each cultivar by Tukey's test (p-value <0.05). Means followed by an asterisk are significantly different between control and heat stress conditions in each cultivar by Tukey's test (p-value <0.05).

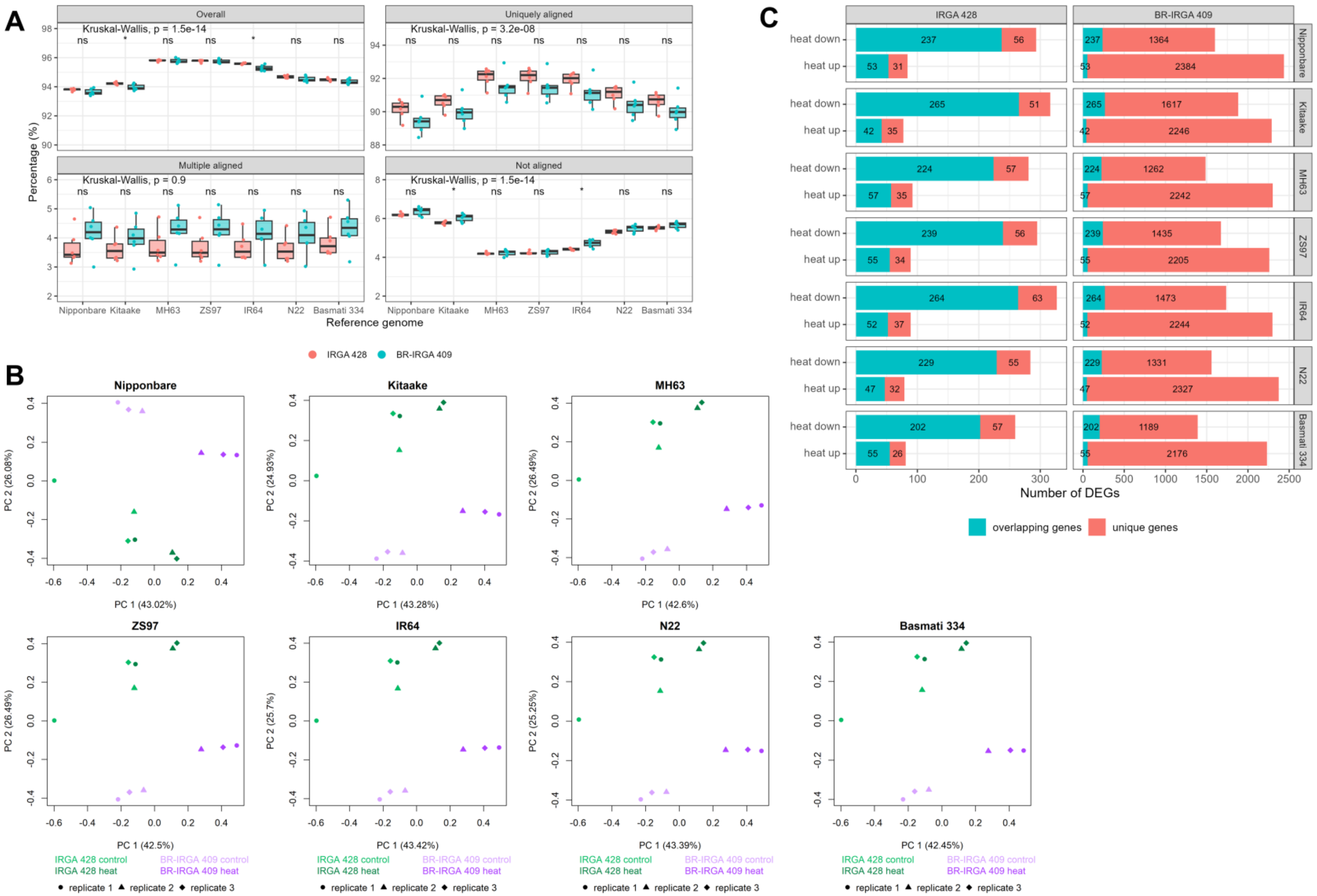

Supplemental Figure 3. (A) Percentage of overall, uniquely aligned, multiply aligned, and unaligned reads mapped to seven rice reference genomes using HISAT2. A Kruskal-Wallis test ($p < 0.05$) was used to compare read percentages across reference genomes within each category, and a Wilcoxon test ($p < 0.05$) was employed for pairwise comparisons between two Brazilian rice varieties. (B) Principal component analysis (PCA) plots depicting gene expression profiles of Brazilian rice varieties under normal and heat stress conditions. Counts per million (CPM) values were derived from read count matrices based on seven rice reference genomes. Colors represent different Brazilian rice varieties, with light and dark shades indicating control and heat stress conditions, respectively. Point shapes denote three biological replicates. (C) Number of heat downregulated and upregulated genes ($log_2FC \geq 0.5$ and $FDR < 0.05$) identified in IRGA 428 and BR-IRGA 409 based on gene read counts derived from seven rice reference genomes. Bar colors indicate overlapping genes shared between the two Brazilian varieties and genes unique to each variety.

Supplemental Figure 4. (A) UpSet plot showing the total number of identified genes shared across five rice reference genomes: Nipponbare, MH63, ZS97, IR64, and N22. (B–C) UpSet plots displaying the distribution of heat downregulated (B) and heat upregulated (C) genes in the IRGA 428 cultivar across the five reference genomes. (D–E) UpSet plots displaying the distribution of heat downregulated (D) and heat upregulated (E) DEGs in the BR-IRGA 409 cultivar across the five reference genomes. Each bar represents the size of the gene intersection for the combination of reference genomes indicated below, while the horizontal bars on the left indicate the total number of genes in each reference genome.

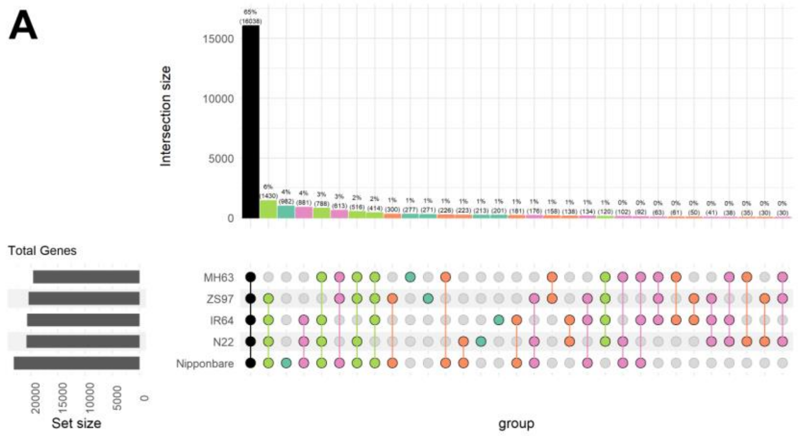

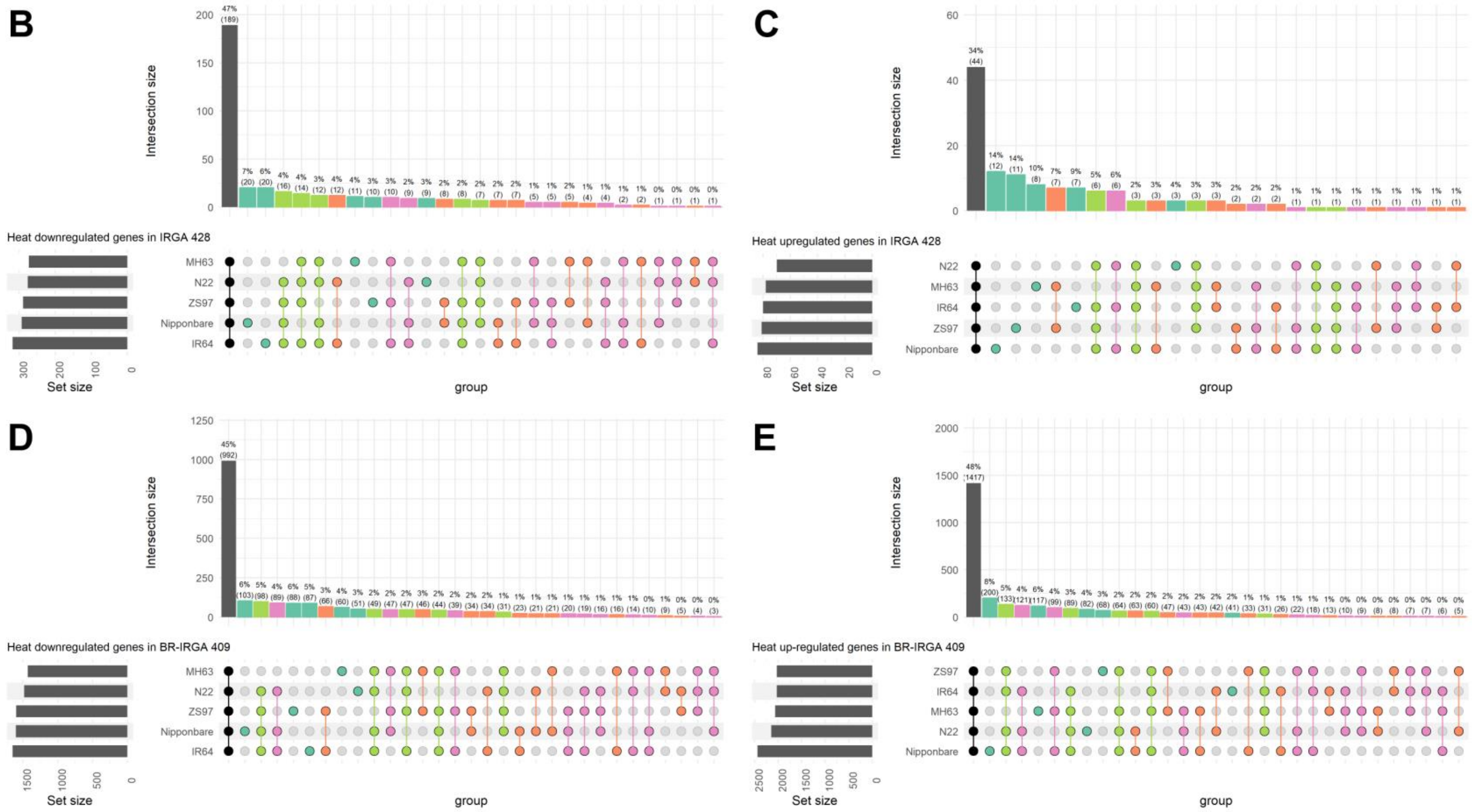

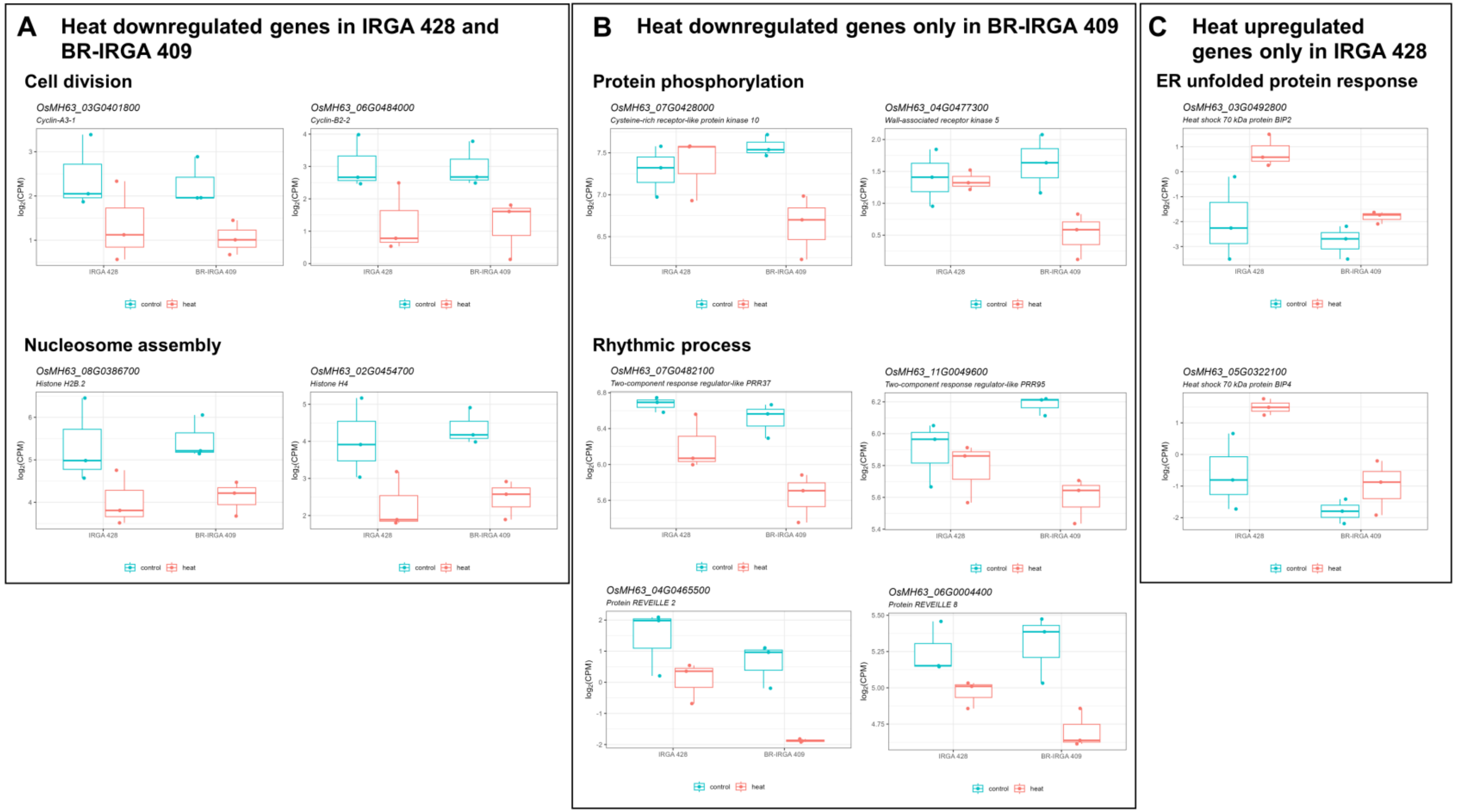

Supplemental Figure 5. (A) Examples of heat downregulated genes in IRGA 428 and BR-IRGA 409 that were present in the 'cell division' and 'nucleosome assembly' GO terms. (B) Examples of heat downregulated genes in BR-IRGA 409 that were present in 'protein phosphorylation' and 'rhythmic process' GO terms. (C) Examples of heat upregulated genes from IRGA 428 that were presented in the 'ER unfolded protein response' GO term.

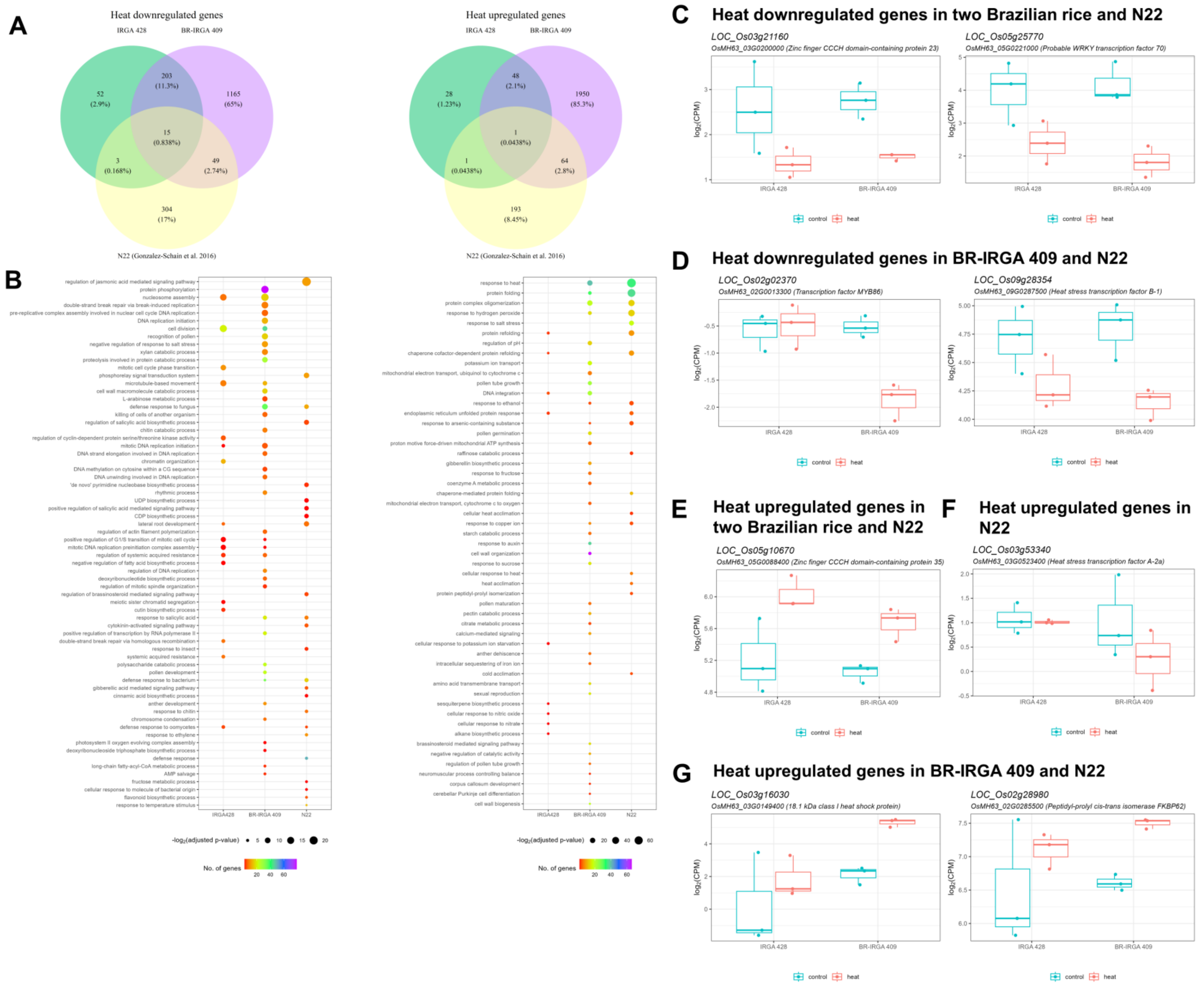

Supplemental Figure 6. (A) Venn diagram comparing the number of heat downregulated and upregulated genes in Brazilian rice and N22 (Gonzales-Schain et al., 2016). (B) Dot plots of significant GO terms (adjusted p-value < 0.05) of heat downregulated (left) and upregulated genes (right) in IRGA 428, BR-IRGA 409, and N22 (González-Schain et al. 2016). (C) Boxplots showing the expression of example genes that were downregulated under heat stress in two Brazilian rice and N22. (D) Boxplots showing the expression of example genes that were downregulated under heat stress in BR-IRGA 409 and N22. (E) Boxplots showing the expression of a gene that was upregulated under heat stress in two Brazilian rice and N22. (F) Boxplot showing the expression of *HSFA2a* (LOC_Os03g53340), the gene that was differentially expressed in N22 (González-Schain et al., 2016), in Brazilian rice. (G) Boxplots showing the expression of example genes that were upregulated under heat stress in BR-IRGA 409 and N22.

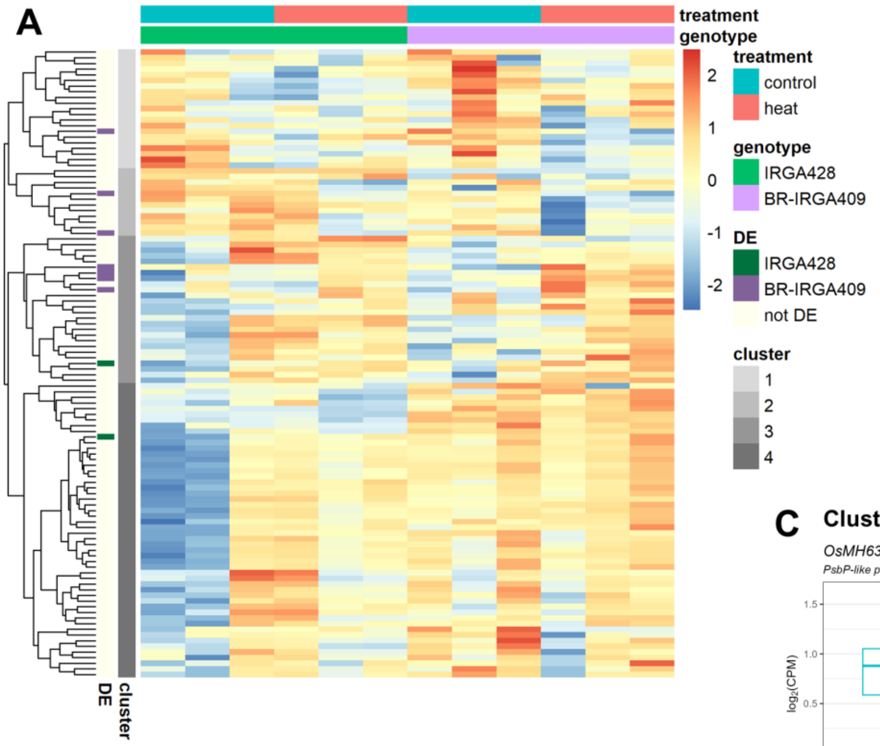

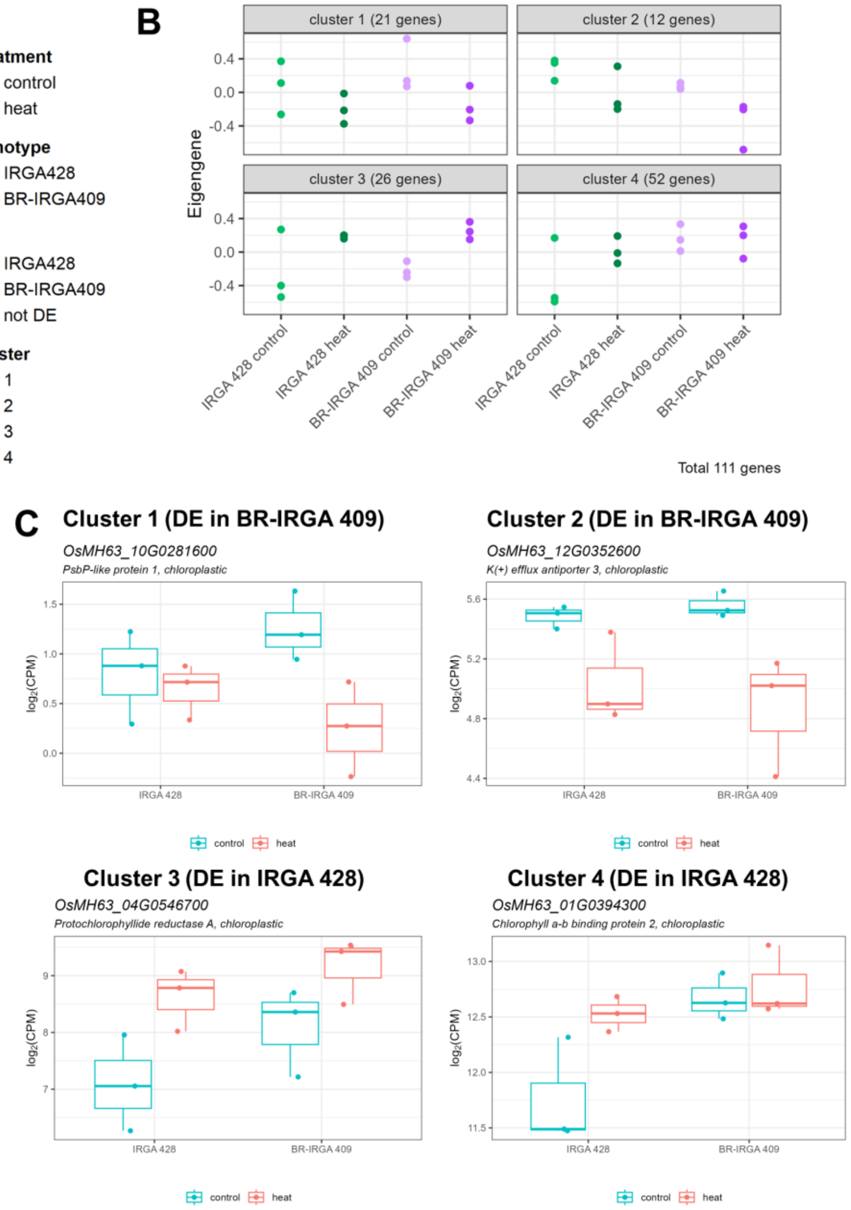

Supplemental Figure 7. Expression of photosynthesis-related genes in Brazilian rice. (A) Heatmaps showing $\log_2$CPM of 111 photosynthesis-related genes in two Brazilian rice varieties under heat stress. Colors in the 'DE' columns indicate whether genes were differentially expressed in IRGA 428 (dark green), BR-IRGA 409 (dark purple), or none (ivory). (B) Eigengenes representing gene expression profiles of DEGs from the interaction effect. (C) Boxplots showing the expression of example genes in each cluster.

Supplemental Table 1. Details of rice reference genomes used in sequence alignment.
Supplemental Table 2. Differentially expressed genes (DEGs) in IRGA 428 under heat stress for three days ($log_2FC \geq 0.5$ and FDR < 0.05).
Supplemental Table 3. Differentially expressed genes (DEGs) in BR-IRGA 409 under heat stress for three days ($log_2FC \geq 0.5$ and FDR < 0.05).
Supplemental Table 4. Biological Gene Ontology (GO) terms associated with genes from the interaction effect between heat stress and cultivar.
Supplemental Table 5. Overlapping heat down-regulated genes between IRGA 428, BR-IRGA 409, and N22 (González-Schain et al. 2016).
Supplemental Table 6. Overlapping heat up-regulated genes between IRGA 428, BR-IRGA 409, and N22 (González-Schain et al. 2016).
